# The *TLR1* gene is associated with higher protection from leprosy in women

**DOI:** 10.1371/journal.pone.0205234

**Published:** 2018-10-05

**Authors:** Eyleen Nabyla Alvarenga Niitsuma, Gabriel da Rocha Fernandes, Francisco Carlos Félix Lana

**Affiliations:** 1 Instituto Federal de Educação, Ciência e Tecnologia do Norte de Minas Gerais, IFNMG, Almenara, Minas Gerais, Brasil; 2 Instituto René Rachou, Fiocruz Minas, Belo Horizonte, Minas Gerais, Brasil; 3 Department of Maternal and Child Nursing and Public Health, Escola de Enfermagem, Universidade Federal de Minas Gerais - UFMG, Belo Horizonte, Minas Gerais, Brasil; Universidade Nova de Lisboa Instituto de Higiene e Medicina Tropical, PORTUGAL

## Abstract

Leprosy is an infectious disease with a complex genetic and immunological background. Polymorphisms in genes that encode cytokines and receptors involved in the immune response, such as the Toll-like receptor 1 (*TLR1*), may be associated with disease risk. We hypothesized that polymorphisms in innate immunity genes confer susceptibility to leprosy that differs between women and men. In this study, we investigate sex differences in the association between a single nucleotide polymorphism (SNP) in *TLR1* and Nucleotide-binding oligomerization domain containing 2 (*NOD2*) genes and leprosy susceptibility in 256 clinically classified leprosy patients and 233 control subjects in a Brazilian population. Our results showed no association between the SNP *rs8057341* in *NOD2* and leprosy in this population. However, the heterozygous genotype of the *TLR1* SNP (*rs4833095*) showed a statistically significant association in women (OR = 0.54, P = 0.02). Our findings suggest that the *TLR1* polymorphism was associated with an increased protection from leprosy in women.

## Introduction

Leprosy is an infectious disease whose etiological agent, *Mycobacterium leprae*, causes cutaneous lesions and impairment of peripheral nerves. The disease is characterized by high infectivity and low pathogenicity [1]. Brazil is the second country with the highest number of cases and higher detection rate in children under fifteen years old in the world [2]. With a broad clinical spectrum, leprosy is distinguished in tuberculoid, borderline tuberculoid, borderline, borderline lepromatous and lepromatous forms [3]. In the tuberculoid form, cell-mediated immunity in the Th1 pattern recruits lymphocytes and natural killer (NK) cells capable of containing the bacillary charge, lead to cure or mild forms of the disease. The lepromatous form, otherwise, has a Th2 pattern with the release of cytokines typical of humoral immunity such as interleukin (IL) 4, IL-5 and IL-10, stimulating the production of antibodies that inhibit the bacillary destruction and contributes to infection progression [4]. The instability between Th1 and Th2 responses is observed in borderline forms [5].

The physiological mechanisms that determine the clinical spectrum are not completely known; therefore, the hypothesis of a genetic component arises to improve understanding of the immune responses precipitated by infection. We know that leprosy susceptibility involves major genes with reduced penetrance and phenotypic expression influenced by minor genes where mutations can alter the functionality of cytokines and receptors [6]. A linkage study in Vietnam identified the association of variants in linfotoxin-α gene and the risk of leprosy, especially in young populations [7]. The infection susceptibility is conducted by initial mechanisms of interaction between the bacillus and macrophages and Schwann cells, which express the pattern recognition receptors (PRRs). These PRRs recognize pathogens and induce signaling cascades that activate genes that encode cytokines, chemokines and costimulatory molecules necessary for adaptive immune responses. Toll-like receptors (TLRs) and Nucleotide oligomerization domain (NOD) like receptors are PRRs [8, 9]. The *I602S* variant in the Toll-like receptor 1 (*TLR1*) gene was shown to be associated with a decreased leprosy incidence in Turkey [10]. A similar result was also found in India [11], but no association was found in Brazilians [12].

Located on chromosome four (4p14), the *TLR1* gene encodes the receptor that recognizes glycolipids and lipopolysaccharides of *M*. *leprae* and activates the transcription factor nuclear factor-kappa B (NF-kβ), which increases the expression of pro-inflammatory genes [13]. Like *TLR1*, nucleotide-binding oligomerization domain containing 2 (*NOD2*) receptor recognizes bacterial peptidoglycans in the intracellular space and activates nuclear factor- kβ (NF-kβ), mitogen-activated protein kinase (MAPK) and interferon regulatory factors (IFN) [14]. The leucine-rich repeat kinase 2 (LRRK2) and receptor-interacting protein kinase 2 (RIPK2) variants are related to a higher risk to develop the disease in Indian populations [15]. Despite the identification of susceptibility genes, the genetics of leprosy and its clinical forms remain complex and still without complete elucidation, reinforcing the need for new studies that can clarify the host genetic profile and interactions established between the immune system and pathogen.

Differences in genetic susceptibility according to sex have already been reported in scientific studies [16, 17], including infectious diseases [18]. Leprosy affects men and women differently with a higher predisposition to tuberculoid forms in women and lepromatous forms in men [19]. However, the effect modification of this variable has not been elucidated given the lack of sex-stratified genetic association studies. We hypothesize that variants in *TLR1* and *NOD2* genes increase leprosy susceptibility by alterations in pathogen recognition, signaling and pro-inflammatory molecule coding, although these mechanisms may differ between sexes. A study showed that polymorphisms in the *TLR1* gene changed the levels of tumor necrosis factor (TNF) and IL-10 in macrophages and were associated with a higher risk for developing leprosy in India and Brazil [20]. Another study demonstrated that *NOD2*-deficient mice failed to produce pro-inflammatory cytokines and were more likely to develop mycobacteria infections [21]. Here, we investigated the association of single nucleotide polymorphisms (SNPs) in *TLR1* (*rs4833095*) and *NOD2* (*rs8057341*) genes with susceptibility to leprosy and reported the first association between a genetic variant in *TLR1* and sex-specific protection against leprosy.

## Materials and methods

### Subjects and study design

We performed a case-control study with leprosy patients and household contacts from the Microregion of Almenara-Minas Gerais, southeast Brazil. Subject recruitment and data collection were obtained from 2011 to 2014. The cases were selected from individuals over seven years old, with a diagnosis based on clinical parameters, skin smear and skin biopsy, registered in the Notifiable Diseases Information System (SINAN) database between 2011 and 2014. State Coordination of Sanitary Dermatology of the Minas Gerais State Health Secretariat, Brazil, made the database available. We adopted the operational classification of leprosy from the World Health Organization (WHO) that classifies leprosy patients as paucibacillary (PB) or multibacillary (MB). We selected household contacts from those who lived with a leprosy patient in the same residence on the date of diagnosis or up to five years before, were over seven years of age, without a diagnosis of leprosy or consanguinity with the case. The exclusion criteria applied to the control group included suspicious clinical manifestations observed in dermato-neurological examination such as dermatological symptoms, palpable thickened nerve or nerve pain, decreased muscle strength and deficit on sensory testing. The study sample consisted of 256 leprosy cases and 233 controls.

Data were collected by home visits, previously authorized, where semi-structured questionnaires specific for cases or controls were applied. These questionnaires had sociodemographic, health and epidemiological questions (the questionnaires are available in the S1 Appendix). Specific questions about the clinical picture were obtained from SINAN consultation. Samples of four milliliters (ml) of blood were collected using an EDTA vacuum system and were stored at -20°C (degrees Celsius). We classified the ethnicity of the cases and controls according to the morphological characteristics as Black, white, indigenous or mestizos. The general sociodemographic characteristics of the study population and the operational classification of the cases are available in the S1 Table.

### Ethical aspects

This study was approved by the Research Ethics Committee of the Federal University of Minas Gerais (COEP-UFMG) in 2010 through the ethics opinion ETIC N°454/2010 and followed the determinations of Resolution 466/2012 of the National Health Council that regulates research with human beings. All subjects were informed about the research objectives, the steps of data collection and the guarantee of anonymity and, subsequently, invited to participate voluntarily. Written informed consent was obtained from all individual participants who agreed to participate. Minors under 18 years of age participated only with verbal and written permission from their parents or guardians.

### Genotyping

Deoxyribonucleic acid (DNA) was extracted from blood samples using the Qiagen Flexigene Kit (category 51206), following the protocol specified by the manufacturer (available at dx.doi.org/10.17504/protocols.io.se4ebgw). The genotyping involved the allelic discrimination through DNA fragment amplification in real-time polymerase chain reaction (RT-PCR) performed using the 7500 *Real Time PCR System* and the SNP genotyping assay for the pre-drawn SNPs *rs4833095* and *rs8057341* with two primers each and two probes corresponding to the SNP of interest. The reaction volume was 20 μl with 20 ng of genomic DNA and 1× reaction buffer composed of Master Mix, and the genotyping assay was performed whose PCR parameters were: 10 minutes at 95 °C for polymerase activation, 50 cycles of 15 seconds at 95 °C for denaturation and 50 cycles of 1 minute at 60 °C for annealing (complete protocol is available at dx.doi.org/10.17504/protocols.io.sfzebp6).

### Data analysis

The univariate analysis used the *odds ratio* and its 95% confidence interval. The *odds ratio* was cauculated from the distribution of genotypes observed between cases and controls. The analysis of subgroup according to the operational classification was performed by the comparison of multibacillary and paucibacillary leprosy cases. To control confounding effects, we performed multivariate analysis by age-adjusted and sex-stratified logistic regression, conducted in software R for Windows [22], version 3.0.0, with the package SNPassoc. Deviations from the expected genotypic frequency were evaluated for the Hardy-Weinberg Equilibrium. All tests assumed a level of statistical significance of a P value < 0.05.

## Results

The frequencies of the expected and observed values for the genotypes and alleles of the SNPs studied were in Hardy-Weinberg equilibrium (P > 0.05). The frequency distributions of alleles and genotypes of the SNPs *rs4833095* of the *TLR1* gene and *rs8057341* of the *NOD2* gene for cases and controls are shown in Table 1.

**Table 1:** Allelic and genotypic frequencies in cases, according to operational classification, and controls.

Locus	Cases	Controls	OR^a^ (95% CI^b^)	PB^c^	MB^d^	MB^d^ vs. PB^c^ OR^a^(95% CI^b^)
	n = 234(%)	n = 233(%)		n = 70(%)	n = 153(%)	
***TLR1_rs4833095***						
*C*	221(47.2)	223(47.8)		65(46.4)	146(47.7)	
*T*	247(52.8)	243(52.2)	1.03 (0.79–1.33)	75(53.6)	160(52.3)	0.95(0.64–1.42)
*C/C*	46(19.7)	58(24.9)	1.52 (0.96–2.42)	12(17.1)	32(20.9)	0.75(0.35–1.61)
*C/T*	129(55.1)	107(45.9)	0.72(0.47–1.11)	41(58.6)	82(53.6)	1.15(0.58–2.27)
*T/T*	59(25.2)	68(29.2)	1.09 (0.65–1.84)	17(24.3)	39(25.5)	0.86(0.36–2.06)
***NOD2_rs8057341***	n = 253(%)	n = 228(%)		n = 81	n = 161	
*A*	159(31.4)	145(31.8)		55(34.0)	101(31.4)	
*G*	347(68.6)	311(68.2)	0.98(0.75–1.29)	107(66.0)	221(68.6)	0.89(0.60–1.33)
*A/A*	30(11.9)	28(12.3)	0.96 (0.54–1.71)	7(8.7)	22(13.7)	1.26(0.49–3.24)
*A/G*	99(39.1)	89(39.0)	0.96(0.53–1.74)	41(50.6)	57(35.4)	2.26(0.88–5.79)
*G/G*	124(49.0)	111(48.7)	0.99(0.68–1.46)	33(40.7)	82(50.9)	0.56(0.32–0.99)*

* Significant values;

^a^ OR: *odds ratio*;

^b^ 95% CI: 95% confidence interval;

^c^ PB: paucibacillary leprosy;

^d^ MB: multibacillary leprosy.

Among the cases and controls, the frequencies of the polymorphic alleles for the SNPs *rs4833095* and *rs8057341* showed very similar proportions. The allelic frequency of *rs4833095* suggests that the *C* ancestral allele was most represented in the control group while the polymorphic *T* allele was most commonly found in leprosy cases.

In order to verify the association of polymorphisms in *TLR1* and *NOD2* genes with leprosy a comparison of allele and genotype frequencies was performed. The comparison was made between cases of leprosy and healthy controls, and between paucibacillary and multibacillary cases in a general model, in which no inheritance was assumed in particular, and in an age-adjusted and stratified by sex model. The unadjusted analysis of the genotypes showed that the homozygous *CC* and *TT* and the heterozygous were not associated with leprosy. The adjusted and stratified by sex distribution of the genotypes showed that the most frequent genotype was the *CT* heterozygous genotype among men and women, but a greater frequency was found in females (61.2%) than in males (50.4%) (Table 2). The analysis under the dominance model showed that the association of the heterozygous genotype with leprosy protection was significant in females (OR = 0.54, 95% CI = 0.32–0.91, P = 0.02) but not in males (OR = 0.83; 95% CI = 0.49–1.41, P = 0.48). Aditional data related to the dominance analysis are given in S2 Table.

**Table 2:** Genotypic frequency of *rs4833095* and *rs8057341* polymorphisms according to sex.

Locus	Cases	Controls	OR^a^ (95% CI^b^)	Cases	Controls	OR^a^ (95% CI^b^)
	Male			Female		
***TLR1_rs4833095***	n = 131(%)	n = 92(%)		n = 103(%)	n = 141(%)	
*C/C*	31(23.7)	23(25)	0.83 (0.49–1.41)	15(14.6)	35(24.8)	0.54 (0.32–0.91)*
*C/T*	66(50.4)	42(45.7)		63(61.1)	65(46.1)	
*T/T*	34(25.9)	27(29.3)		25(24.3)	41(29.1)	
***NOD2_rs8057341***	n = 139(%)	n = 89(%)		n = 114(%)	n = 139(%)	
*A/A*	18(12.9)	9(10.1)	0.51 (0.32–1.77)	12(10.5)	19(13.7)	0.62 (0.45–2.90)
*A/G*	46(33.1)	33(37.1)		53(46.5)	56(40.3)	
*G/G*	75(54.0)	47(52.8)		49(43.0)	64(46)	

* Significant values;

^a^ OR: *odds ratio* age-adjusted and under dominance model;

^b^ 95% CI: 95% confidence interval.

The distribution of alleles of SNP *rs8057341* in the *NOD2* gene showed that the polymorphic *A* allele was infrequent in the cases and controls. The *AA* and *AG* genotypes were also poorly represented in the study population compared with the homozygous genotype for the ancestral allele, but this difference between cases and controls was not statistically significant in the unadjusted analysis (Table 1). The adjusted sex-stratified analysis showed that male gender predominated for the homozygous *GG* genotype; however, in females, there was an equivalent distribution of the *GG* and *AG* genotypes. The genotypic frequencies in men and women between cases and controls showed no significant difference. Both the genotypic frequencies and sex-stratified analysis of *rs4833095* and *rs8057341* with leprosy can be seen in Table 2.

For *rs4833095*, the allele proportion between the paucibacillary and multibacillary cases was quite close as well as the proportion of the genotypes. The polymorphic allele in *rs8057341* was less frequent in both leprosy operational classifications and was found in approximately 30% of the cases. In paucibacillary cases, *AG* heterozygous and *GG* homozygous were predominant with a low frequency of the polymorphic allele. This genotype distribution was similar in multibacillary cases. Analysis showed no association of the SNP in *TLR1* gene with risk or protection for any clinical form of leprosy. However, in the analysis of *NOD2* gene, the difference of the genotypic frequencies of the *AG* heterozygous with the homozygous *GG* between paucibacillary and multibacillary cases showed a borderline statistical difference (OR = 0.56, 95% CI = 0.32–0.99, P = 0.046). Stratification by sex was not possible due to the consequent sample reduction and lack of statistical support for the analysis.

## Discussion

In this study, we hypothesized that variants in the *TLR1* and *NOD2* genes alter the recognition of the pathogen and activate the transcription factor NF-kβ, increasing the expression of pro-inflammatory molecules [13, 14]. A scientific study conducted in Minas Gerais, Brazil showed that the variant *rs4833095* in the *TLR1* gene is a protective factor for leprosy.

The frequency of the *rs4833095* polymorphic allele in the Americas is well approximated to the proportion observed in this study sample (52 and 48%, respectively) [23]. A case-control study conducted in India found a higher frequency of the *T* allele among cases of leprosy in the proportion of 54% for cases and 51% for controls (OR = 1.12, 95% CI = 0.97–1.31) similar to the results found in this study, where the *T* was more frequent between the cases (75 and 72%) [20].

The genotype frequency in American populations had the following distribution: 47.3% heterozygous, 23.6% homozygous for the ancestral allele and 29.1% homozygous for the polymorphic allele [23]. Although the heterozygous frequency in the Americas was equivalent to this sample, the homozygous genotypes for the ancestral allele and polymorphic allele were inversely proportional in both populations (27.2 and 22.3%). In the Indian population, *TT* was associated with leprosy susceptibility per se (OR = 1.4, 95% CI = 1.06–1.70), the heterozygous showed a protective effect (OR = 0.78, 95% CI = 0.63–0, 96) and *CC* displayed no association [20]. In a case-control study in São Paulo, Brazil, *TT* and *CT* were associated with leprosy susceptibility per se (OR = 1.58, 95% CI = 1.10–2.24, P = 0.01 and OR = 1.81, 95% CI = 1.20–2.71, P = 0.004). The replication of this study in other populations of Brazil (Rondonópolis, Mato Grosso and Rio de Janeiro) obtained similar results [12].

The *rs4833095* involves the substitution of a guanine for an adenine in the DNA sequence and is designated N248S. It is a missense variant that results in the change of asparagine to serine at position 248 of the protein, decreasing TRL1 receptor expression in the cells of the immune system [24]. Commonly found in African-Americans, this SNP was strongly represented in tuberculosis cases [25] and is associated with pulmonary and cardiovascular pro-inflammatory complications in septicemia and multiple organ dysfunction [26]. A study conducted with individuals of various ethnicities found that *rs4833095* impaired the release of cytokines by monocytes, predisposing to *Candida albicans* infection in the Caucasian population [27]. The polymorphic allele was also associated with the reverse reaction in leprosy patients in India [20].

The amino acid 248 of *TLR1* is located at the external binding site, and individuals with the *T* allele have been shown to have reduced expression of *TLR1* on the surface of leukocytes. It is possible that a defect in cellular traffic will impair the transport of the receptor to the cell membrane, altering its expression and recognition of *M*. *leprae* [24]. Analysis of the cytokine levels of cells from healthy subjects found that, when stimulated by *M*. *leprae* antigens, the ratio of the level of TNF to the level of IL-10 was significantly lower in those with the *T* allele. The release of TNF and its role in containing bacillary load and progression to spontaneous cure or to disease with milder manifestations may be inhibited in individuals with the polymorphic allele [12]. However, as evidenced by the results of this study, complete dominance in heterozygous individuals may help to understand how heterozygosity can suppress the detrimental effect of the polymorphic allele and confer leprosy *per se* protection.

The present study is a pioneer in pointing out the protection to leprosy conferred by the *C* allele of the *TLR1* gene in the female sex, evidenced by the statistically significant association. In Brazil, the detection coefficients between the sexes from 2001 to 2007 were 28.94 / 100.000 for males and 22.63 / 100.000 for females [28]. In leprosy, as in other infectious diseases, the differences in incidence between the sexes are contrasting. The hypothesis is that sex hormones interact with the immune system and precipitate differences in susceptibility between men and women with estrogen upregulation and the down-regulation of immune responses by testosterone [19], influencing the transcription of several genes [29].

A study showed that a higher production of IFN-α under TLR7 stimulation in dendritic cells occurs in females [30], a finding that was repeated when the stimulated receptor was TLR9. Although the production of IFN-α and TNF-α appears to be higher among women, only IFN-α has been shown to be significant so far [18]. The genes in the sex chromosomes also seem to affect the development of diseases, especially autoimmune ones, through the low production of Th2 cytokines by the macrophages and dendritic cells of the female mouse [31]. Cellular immunity can be considered a protective factor for leprosy due to the control it exerts in the bacillary load, different from humoral immunity, which is common in the most severe forms of the disease. The *CT* heterozygous women appear to produce more pro-inflammatory molecules when the *TLR1* gene is activated, resulting in a more effective immune response to *M*. *leprae* infection, as hypothesized earlier. This effect can be mediated by sex hormones, genes in sex chromosome and polymorphisms in immune system genes, co-responsible for the mediation of innate and adaptive immune responses. Studies have shown that steroid hormones can bind to specific elements of the hormonal response in the promoter regions of hormone-responsive genes, which may indicate the presence of androgen- and estrogen-response elements in the promoters of various genes of innate immunity [32].

The allelic frequency of *rs8057341* differed from the proportions in the American populations that presented higher frequency of the polymorphic allele (48.4%) [33]. In contrast to other studies conducted in Brazilian populations that verified the association of the *AA* genotype with resistance to leprosy [34], our analysis was not statistically significant, likely explained by the low frequency of the polymorphic allele in the sample. New studies with a larger sample are necessary to detect subtle differences in the allelic and genotypic distribution in this population. However, we must consider the genetic diversity among the populations and contribution of genetic diversity in the differences in susceptibility to infectious diseases [35]. Adjusted analyses were used to minimize the effects of possible confounding variables; in addition, all the subjects of the study were recruited in the same microregion.

We emphasize that the additional inclusion of controls from non-endemic Brazilian areas could also have contributed to the analysis of differences in genetic susceptibility, which will be a starting point for future studies. New studies that include other polymorphisms, such as *I602S* in the *TLR1* gene designated as protector in the populations of Turkey^10^ and India^11^, are needed to improve understanding about genetic susceptibility to leprosy as well as the differences in susceptibility between men and women.

Several studies have already indicated a significant association of *rs4833095* with leprosy; none of them, however, showed a specific association with sex. This study showed that the heterozygous genotype is a specific protection factor for female sex, and the mechanisms and signaling pathways that guide this effect, as well as the role of sex hormones in the protection of leprosy, are still unclear and require further research to be elucidated.

## Supporting information

S1 Table Demographic and clinical characteristics of individuals included in the study. *Number of individuals with available information; ^a^±SD: Standard deviation.
(DOCX)

S2 Table Association of the *rs4833095* and *rs8057341* polymorphisms with leprosy, according to sex. * Significant values; ^a^ OR: *odds ratio*; ^b^ 95% CI: 95% confidence interval.
(DOC)

S1 Appendix Questionnaire for data collection. (PDF)
