# Supplementary material for: The TLR1 gene is associated with higher protection from leprosy in women
Source: PLoS One. 2018 Oct 5;13(10):e0205234. doi: 10.1371/journal.pone.0205234 (PMC6173409; doi:10.1371/journal.pone.0205234)
Supplement: S1 Table — *Number of individuals with available information; a±SD: Standard deviation. (DOCX) [file pone.0205234.s001.docx]

| **Variable** | **Cases** | **Controls** |
| --- | --- | --- |
|  | n = 256 | n = 233 |
| Sex, n* (%) |  |  |
| Male | 142(55.5) | 92(39.5) |
| Female | 114(44.5) | 141(60.5) |
| Age, years mean ± SD^a^ | 48.7 ± 20.1 | 48,9 ± 18.1 |
| Ethnic group, n^*^ (%) |  |  |
| Black | 27(12.7) | 13(6.8) |
| White | 37(17.5) | 39(20.3) |
| Indigenous | 1(0.5) | 1(0.5) |
| Mestizo | 147(69.3) | 139(72.4) |
| WHO Operational classification, n* (%) |  |  |
| Paucibacillary | 82(33.5) |  |
| Multibacillary | 163(66.5) |  |

**S1 Table.** Demographic and clinical characteristics of individuals included in the study.

^*^ Number of individuals with available information. The number reported may be different from the total number of individuals due to missing information.

^a^ *±SD*: Standard deviation.
