# Supplementary material for: The TLR1 gene is associated with higher protection from leprosy in women
Source: PLoS One. 2018 Oct 5;13(10):e0205234. doi: 10.1371/journal.pone.0205234 (PMC6173409; doi:10.1371/journal.pone.0205234)
Supplement: S2 Table — * Significant values; a OR: odds ratio; b 95% CI: 95% confidence interval. (DOC) [file pone.0205234.s002.doc]

| **S2 Table.** Association of the *rs4833095* and *rs8057341* polymorphisms with leprosy, according to sex. | | | | | | | | |
| --- | --- | --- | --- | --- | --- | --- | --- | --- |
| **Locus** | **Cases** | | **Controls** | | **Cases** | | **Controls** | |
|  | Male | | | | Female | | | |
| ***TLR1_rs4833095*** | **OR^a^** | **95% CI^b^** | | **p-value** | **OR^a^** | **95% CI^b^** | | **p-value** |
| Codominant |  |  | |  |  |  | |  |
| *C/C* | Reference |  | | 0.77 | Reference |  | | 0.04 |
| *C/T* | 0,80 | 0.42-1.52 | |  | 0.63 | 0.34-1.15 | |  |
| *T/T* | 0.93 | 0.45-1.96 | |  | 1.42 | 0.65-3.10 | |  |
| Dominant |  |  | |  |  |  | |  |
| *C/C* | 1 |  | |  | 1 |  | |  |
| *C/T-T/T* | 0.84 | 0.47-1.53 | | 0.57 | 0.78 | 0.44-1.39 | | 0.40 |
| Recessive |  |  | |  |  |  | |  |
| *C/C-C/T* | 1 |  | |  | 1 |  | | 0.04 |
| *T/T* | 1.08 | 0.58-2.0 | | 0.81 | 1.93 | 0.99-3.76 | |  |
| Overdominant |  |  | |  |  |  | |  |
| *C/C-T/T* | 1 |  | |  | 1 |  | |  |
| *C/T* | 0.83 | 0.49-1.41 | | 0.48 | 0.54* | 0.32-0.91* | | 0.02* |
|  |  |  | |  |  |  | |  |
| ***NOD2_rs8057341*** |  |  | |  |  |  | |  |
| Codominant |  |  | |  |  |  | |  |
| *A/A* | Reference |  | | 0.73 | Reference |  | | 0.54 |
| *A/G* | 1.14 | 0.64-2.03 | |  | 0.80 | 0.47-1.37 | |  |
| *G/G* | 0.80 | 0.33-1.92 | |  | 1.20 | 0.53-2.72 | |  |
| Dominant |  |  | |  |  |  | |  |
| *A/A* | 1 |  | |  | 1 |  | |  |
| *A/G-G/G* | 1.04 | 0.61-1.78 | | 0.87 | 0.88 | 0.53-1.45 | | 0.60 |
| Recessive |  |  | |  |  |  | |  |
| *A/A-A/G* | Reference |  | |  | Reference |  | |  |
| *G/G* | 0.76 | 0.32-1.77 | | 0.51 | 1.34 | 0.62-2.90 | | 0.45 |
| Overdominant |  |  | |  |  |  | |  |
| *A/A-G/G* | 1 |  | |  | 1 |  | |  |
| *A/G* | 1.19 | 0.68-2.07 | | 0.54 | 0.77 | 0.47-1.28 | | 0.31 |

* Significant values;

^a^ OR: *odds ratio* age-adjusted;

^b^ 95% CI: 95% confidence interval.
