# Supplementary material for: The TLR1 gene is associated with higher protection from leprosy in women
Source: PLoS One. 2018 Oct 5;13(10):e0205234. doi: 10.1371/journal.pone.0205234 (PMC6173409; doi:10.1371/journal.pone.0205234)
Supplement: S1 Appendix — (PDF) [file pone.0205234.s003.pdf]

S1 Appendix. Questionnaire for data collection

Leprosy patient questionnaire

Identification		
Interview date: / /	Interviewer:	City:
Health unit:		
Name:		Nickname:
Current address:		
e-mail:		
Age:	Date of birth: / /	Sex: ( ) Male ( ) Female
Ethnic group: ( ) Black ( ) White ( ) Indigenous ( ) Mestizo		
City of birth:		State:
Someone who resided with you up to 5 years before you had leprosy moved?<br>( ) No ( ) Yes. How many people?		
Epidemiological data		
Any other residents have or had leprosy? ( ) No ( ) Yes. How many?		
Besides leprosy, you had or have another disease?<br>( ) No<br>( ) Yes. Specify:		
Are you pregnant? ( ) No ( ) Yes ( ) Not applicable<br>What is your gestational age?		
Do you suspect pregnancy?		
Do you use any medication regularly?<br>( ) No ( ) Yes. Specify:		
Clinical information		
Registration number in SINAN:		
Treatment start date: / /	End date of treatment:	Therapeutic scheme:
Operational classification: ( ) Paucibacillary ( ) Multibacillary		

Household contact questionnaire

Identification		
Interview date: / /	Interviewer:	City:
Health unit:		
Name:		Nickname:
Current address:		
e-mail:		
Age:	Date of birth: / /	Sex: ( ) Male ( ) Female
Ethnic group: ( ) Black ( ) White ( ) Indigenous ( ) Mestizo		
City of birth:		State:
**Epidemiological data**		
Have you ever had leprosy? ( ) No ( ) Yes		
Do you had or have another disease? ( ) No ( ) Yes. Specify:		
Are you pregnant? ( ) No ( ) Yes ( ) Not applicable<br>What is your gestational age?		
Do you suspect pregnancy?		
**Dermatoneurological evaluation**		
Suggestive signs of leprosy? ( ) No ( ) Yes. Specify:		
Dermatological evaluation	Neurological evaluation	Sensitivity test
Skin lesions?<br>( ) No ( ) Yes How many?<br><br>Skin lesions with sensory loss?<br>( ) No ( ) Yes How many?<br><br>Skin plaques?<br>( ) No ( ) Yes How many?<br><br>Tubercles?<br>( ) No ( ) Yes How many?<br><br>Nodules?<br>( ) No ( ) Yes How many?<br><br>Infiltration?<br>( ) No ( ) Yes How many?<br><br>Others?<br>( ) No ( ) Yes How many?	Thickened nerves?<br>( ) No ( ) Yes.<br><br>Specify:<br>( ) Ulnar<br>( ) Radial<br>( ) Lateral popliteal<br>( ) Posterior tibial<br><br>Reduced muscle strenght<br>( ) No ( ) Yes.<br><br>Specify:<br>( ) Eyelids<br>( ) Abductor of the fifth finger<br>( ) Abductor of the thumb<br>( ) Wrist extensor<br>( ) Halux extensor<br>( ) Long extensor of toes<br>( ) Tibialis anterior muscle<br>( ) Peroneous muscles	Sensory deficit?<br>( ) No ( ) Yes.<br><br>Specify:<br>( ) Left eye<br>( ) Right eye<br>( ) Left hand<br>( ) Right hand<br>( ) Left foot<br>( ) Right foot
Confirmed diagnosis? ( ) No ( ) Yes ( ) Not evaluated ( ) Not applicable		
**Information related to the case**		
Degree of kinship:		
